# Tamoxifen-predictive value of gene expression signatures in premenopausal breast cancer: data from the randomized SBII:2 trial

**DOI:** 10.1186/s13058-023-01719-z

**Published:** 2023-09-29

**Authors:** Christine Lundgren, Julia Tutzauer, Sarah E. Church, Olle Stål, Maria Ekholm, Carina Forsare, Bo Nordenskjöld, Mårten Fernö, Pär-Ola Bendahl, Lisa Rydén

**Affiliations:** 1 Department of Oncology, Region Jönköping County, Jönköping, Sweden; 2 Department of Biomedical and Clinical Sciences, Linköping University, Linköping, Sweden; 3 Division of Oncology, Department of Clinical Sciences Lund, Lund University, Medicon Village, Building 404, 223 81 Lund, Sweden; 4 NanoString Technologies, Inc., Seattle, USA; 5 Division of Surgery, Department of Clinical Sciences Lund, Lund University, Lund, Sweden; 6 Department of Surgery, Skåne University Hospital, Malmö, Sweden

**Keywords:** Gene expression signatures, Premenopausal, Tamoxifen, Prognostic, Predictive

## Abstract

**Background:**

Gene expression (GEX) signatures in breast cancer provide prognostic information, but little is known about their predictive value for tamoxifen treatment. We examined the tamoxifen-predictive value and prognostic effects of different GEX signatures in premenopausal women with early breast cancer.

**Methods:**

RNA from formalin-fixed paraffin-embedded tumor tissue from premenopausal women randomized between two years of tamoxifen treatment and no systemic treatment was extracted and successfully subjected to GEX profiling (*n* = 437, NanoString Breast Cancer 360™ panel). The median follow-up periods for a recurrence-free interval (RFi) and overall survival (OS) were 28 and 33 years, respectively. Associations between GEX signatures and tamoxifen effect were assessed in patients with estrogen receptor-positive/human epidermal growth factor receptor 2-negative (ER+ /HER2−) tumors using Kaplan–Meier estimates and Cox regression. The prognostic effects of GEX signatures were studied in the entire cohort. False discovery rate adjustments (*q*-values) were applied to account for multiple hypothesis testing.

**Results:**

In patients with ER+/HER2− tumors, *FOXA1* expression below the median was associated with an improved effect of tamoxifen after 10 years with regard to RFi (hazard ratio [HR]_*FOXA1*(high)_ = 1.04, 95% CI = 0.61–1.76, HR_*FOXA1*(low)_ = 0.30, 95% CI = 0.14–0.67, *q*_interaction_ = 0.0013), and a resembling trend was observed for *AR* (HR_*AR*(high)_ = 1.15, 95% CI = 0.60–2.20, HR_*AR*(low)_ = 0.42, 95% CI = 0.24–0.75, *q*_interaction_ = 0.87). Similar patterns were observed for OS. Tamoxifen was in the same subgroup most beneficial for RFi in patients with low *ESR1* expression (HR_RFi *ESR1*(high)_ = 0.76, 95% CI = 0.43–1.35, HR_RFi, *ESR1*(low)_ = 0.56, 95% CI = 0.29–1.06, *q*_interaction_ = 0.37). Irrespective of molecular subtype, higher levels of *ESR1*, Mast cells, and *PGR* on a continuous scale were correlated with improved 10 years RFi (HR_*ESR1*_ = 0.80, 95% CI = 0.69–0.92, *q* = 0.005; HR_Mast cells_ = 0.74, 95% CI = 0.65–0.85, *q* < 0.0001; and HR_*PGR*_ = 0.78, 95% CI = 0.68–0.89, *q* = 0.002). For BC proliferation and Hypoxia, higher scores associated with worse outcomes (HR_BCproliferation_ = 1.54, 95% CI = 1.33–1.79, *q* < 0.0001; HR_Hypoxia_ = 1.38, 95% CI = 1.20–1.58, *q* < 0.0001). The results were similar for OS.

**Conclusions:**

Expression of *FOXA1* is a promising predictive biomarker for tamoxifen effect in ER+/HER2− premenopausal breast cancer. In addition, each of the signatures BC proliferation, Hypoxia, Mast cells, and the GEX of *AR*, *ESR1,* and *PGR* had prognostic value, also after adjusting for established prognostic factors.

*Trial registration* This trial was retrospectively registered in the ISRCTN database the 6th of December 2019, trial ID: https://clinicaltrials.gov/ct2/show/ISRCTN12474687.

**Supplementary Information:**

The online version contains supplementary material available at 10.1186/s13058-023-01719-z.

## Background

Although endocrine therapy with tamoxifen significantly reduces the risk of recurrence in patients with estrogen receptor-positive (ER+) breast cancer, breast cancer recurrence 20 years after diagnosis is not uncommon [1]. Moreover, some patients with ER+ tumors do not benefit from this treatment [2, 3]. Despite this, ER status is the only clinically established predictive marker for tamoxifen response [4], highlighting the need for new predictive tools. In patients treated with five years of adjuvant endocrine therapy, the risk of recurrence is strongly correlated with tumor size, nodal status, and histological grade [1]. Furthermore, the progesterone receptor (PR) has been observed to be prognostic [5], but its independent predictive effect on the response to endocrine therapy has not been established [6]. In recent decades, the clinical use of gene expression (GEX) analysis for prognostication has increased. In addition to providing information on intrinsic subtypes, GEX signatures have been observed to add putative predictive value [7–11], even for late recurrences [12]. However, the use of risk scores in premenopausal patients is not widely implemented [11, 13].

In addition to routine markers, GEX may provide additional information for predicting the effects of breast cancer drugs [14–16]. This was exemplified in the FinXX trial using the NanoString Breast Cancer (BC) 360™ panel (BC360 panel), where cytotoxic, endothelial, and Mast cell GEX signatures predicted improved recurrence-free survival, favoring the addition of capecitabine to adjuvant chemotherapy in patients with triple-negative breast cancer (TNBC) [15]. Previously, we demonstrated that PAM50 luminal subtypes are associated with the efficacy of adjuvant tamoxifen in premenopausal patients [9]; however, other gene signatures are currently not used in clinical practice to guide the use of endocrine therapy. The *ESR1* gene encodes ER alpha (ERα, denoted as ER in this manuscript), and the GEX of *ESR1* and protein expression of ER are strongly correlated [17]. Therefore, high *ESR1* GEX levels could indicate responsiveness to tamoxifen therapy, as demonstrated by Chungyeul et al.; however, the same effect was not observed for *PGR* GEX [16]. Although GEX levels of the androgen receptor (*AR*) seem to be associated with better outcome [18], and *AR* overexpression has been reported to induce tamoxifen resistance in a preclinical setting [19], no clear endocrine-predictive effect has been observed [20].

Despite comprehensive studies on GEX signatures in relation to prognosis and prediction of treatment response in primary breast cancer, only a few have been used in the clinical setting. High proliferation scores including Oncotype DX, Prosigna gene assay, and hypoxic GEX signature have been associated with a worse prognosis [21–24]. In contrast, high expression of the *FOXA1* gene seems to be associated with better outcomes in patients with ER+ breast tumors [25, 26].

Previously, we reported the long-term effects of tamoxifen and prognostic value of PAM50 subtypes and the risk of recurrence (ROR) score based on the BC360 panel for premenopausal patients who were randomized between two years of adjuvant tamoxifen and no systemic treatment in the SBII:2pre trial [9]. The primary aim of the present study was to determine the tamoxifen-predictive value of GEX signatures from the BC360 Panel with respect to recurrence-free interval (RFi) and overall survival (OS) in patients with ER+/human epidermal growth factor receptor 2-negative (ER+/HER2 −) tumors. The secondary aim was to decipher the prognostic value of the signatures regardless of molecular subtype.

## Methods

### Study population

**Fig. 1:**
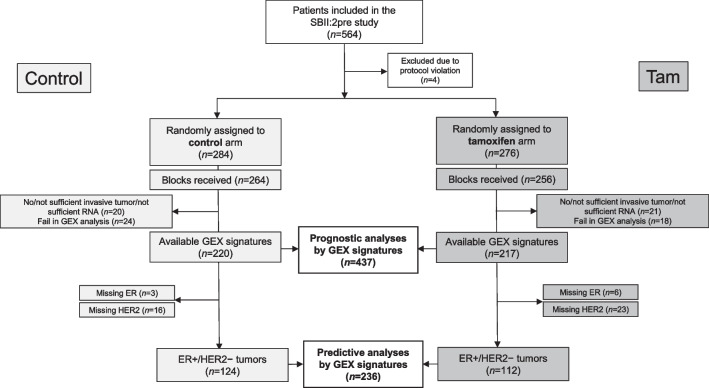
Flowchart of included patients. *ER* estrogen receptor, *GEX* gene expression, *HER2* human epidermal growth factor receptor 2, *n* number of patients, *RNA* ribonucleic acid, *Tam *tamoxifen

A flowchart of the study cohort is shown in Fig. 1. In the SBII:2pre trial, 564 premenopausal women were randomized to receive 2 years of adjuvant tamoxifen or no systemic treatment [9, 27–30]. The translated, abbreviated study protocol is available in Additional file 1, which provides information on the inclusion and exclusion criteria. In this study, treatment-predictive analyses were performed in patients with ER+/HER2− tumors only (*n* = 236), whereas all patients with GEX data (*n* = 437) were included in the prognostic analyses.

### Study endpoints and follow-up data

The endpoints were RFi (including any of the following first events: invasive ipsilateral breast cancer recurrence and ductal cancer in situ; local, regional, or distant recurrence; or breast cancer-related death) and OS. The data cutoff for RFi was November 30, 2016. OS data were retrieved from the Swedish Causes of Death Register (data cutoff for events was December 10, 2020). Endpoints were defined according to DATECAN recommendations [31]. Results were reported for the maximum follow-up and, because of non-proportional hazards, also for the time interval of 0–10 years.

### Tumor characteristics and GEX signatures

Archived formalin-fixed paraffin-embedded (FFPE) breast tumor tissues from *n* = 520 of the study participants were collected. Methods for RNA extraction and assessment of ER, Ki67, PR, histological grade (Nottingham histological grade [NHG]), HER2, and stromal tumor-infiltrating lymphocytes (sTILs, here denoted TILs) have been published [9]. GEX analysis was performed according to the manufacturer’s instructions using a NanoString BC360™ panel [32]. This panel included 776 genes and the calculated scores of a panel of GEX signatures in breast cancer (Additional file 2). The BC360 panel included 48 GEX signatures, of which 18 were single genes (Additional file 2). Raw data were normalized on a log2 scale using housekeeping genes and BC360 panel standards. In total, 91% (437) of the 479 samples with sufficient amount of invasive tumor tissue and extracted RNA passed the quality control check.

### Selection of gene signatures

The prognostic and predictive effects were analyzed for 41 GEX signatures selected from the BC360 panel. For the detailed predictive analyses, we selected the *ESR1*, which is known to be of importance for endocrine resistance [16, 17] and *PGR*, which is closely related to *ESR1*. Furthermore, we selected BC360 panel signatures based on their relationships with the outcomes used in this study, as visualized in the forest plots. We excluded the subtype signatures of PAM50 (Luminal A, Luminal B, HER2-enriched (HER2-E), and basal-like) and ROR from the prognostic and predictive screening, as these data have been previously reported for this trial [9]. Additionally, we excluded the genomic risk signature, as this is ROR without accounting for tumor size, and the TNBC subtype signatures as TNBC comprised only a minority of the samples, and luminal tumors were the focus of the study. However, the PAM50 subtypes were included in the multivariable analyses. Only the abbreviated names of the GEX signatures are used in this report; the abbreviations can be found in the abbreviation list.

### Statistical analyses

RStudio using R version 4.2.2 was used for all the statistical analyses and all the tests were two-sided. To account for multiple hypothesis testing, each set of analyses was adjusted for false discovery rate (FDR) [33]. FDR-adjusted *p*-values are denoted *q*-values, while crude *p*-values are denoted *p*-values, and values < 0.05 were generally considered statistically significant. Unless otherwise stated, the expression of single genes and GEX signatures were normalized using the sample mean and standard deviation (SD) and analyzed as continuous variables [34]. When grouping the cohort based on the GEX data was necessary, this was based on gene signature medians or quartiles.

Associations between the GEX signatures and clinicopathological variables were assessed using Pearson’s correlation and visualized using the R package *corrplot* [35]. To further visualize GEX signature expression across the cohort, a heatmap was constructed using the R package *ComplexHeatmap* [36]. Dendrograms were generated using complete Euclidean hierarchical clustering. K-means clustering was used to detect four clusters among the tumor samples and GEX signatures (20 initializations and random centroids). The number of clusters was selected based on the visual patterns and to optimize the stability of the results.

Cox proportional hazards regression with standardized GEX signatures modeled as continuous variables was used to calculate hazard ratios (HRs). Multivariable Cox models were adjusted for PAM50 subtype, nodal category (positive *vs*. negative), age (continuous), NHG, tumor size (> 20 mm *vs*. ≤ 20 mm), and treatment arm (the latter not included in predictive analyses). The results from the Cox models were visualized in forest plots. The relationship between GEX signatures, tamoxifen treatment, and outcomes was graphically assessed further using Kaplan–Meier curves. Proportional hazard assumptions were graphically verified using Schoenfeld residuals (data not shown) [37]. The proportional hazard assumptions were generally not met. Hazard ratios should therefore be carefully interpreted as average effects over the follow-up period. The tamoxifen-predictive effect of the selected signatures was evaluated using Cox regression with the main effects for treatment, signature, and an interaction term. The interaction term was defined as the product of the continuous GEX signature score and the binary treatment variable.

The results are, where applicable, presented following the Reporting Recommendations for Tumor Marker Prognostic Studies (REMARK) [38, 39].

## Results

### Study cohort characteristics

Tumor blocks from patients in the control and tamoxifen treatment arms were analyzed using the BC360 panel (Fig. 1). Patient and tumor characteristics for the full study cohort with (*n* = 437) and without (*n* = 123) available GEX data by treatment arm are presented in Table 1, and for the ER+/HER2− cohort in Table 2. The median follow-up period for patients without events was 28 years (range; 8–32) and 33 years (range; 11–37) in the prognostic analyses of RFi and OS, respectively.

**Table 1:** Patient and tumor characteristics

Characteristics	Patients with gene signatures (*n* = 437)		Patients without gene signatures (*n* = 123)	
	Control group *n* (%)	Tam-treated group *n* (%)	Control group *n* (%)	Tam-treated group *n* (%)
Age (years)				
Median	45	45	45	47
Range	27–54	26–57	29–58	31–55
Tumor size (mm)				
≤ 20	86 (39)	69 (32)	35 (55)	17 (29)
> 20	134 (61)	148 (68)	29 (45)	41 (71)
Missing	0	0	0	1
Nodal status				
Node-negative	57 (26)	62 (29)	18 (28)	21 (36)
Node-positive	162 (74)	154 (71)	46 (72)	38 (64)
N1	105 (48)	108 (50)	34 (53)	28 (48)
N2	57 (26)	46 (21)	12 (19)	10 (17)
Missing	1	1	0	0
NHG				
1	24 (11)	22 (11)	1 (15)	5 (11)
2	88 (42)	87 (43)	27 (52)	18 (38)
3	99 (47)	93 (46)	17 (33)	24 (51)
Missing	9	15	12	12
ER				
Positive	154 (70)	139 (66)	37 (64)	32 (63)
Negative	63 (29)	72 (34)	21 (36)	19 (37)
Missing	3	6	6	8
PR				
Positive	148 (68)	132 (61)	37 (64)	31 (62)
Negative	71 (32)	84 (39)	21 (36)	19 (38)
Missing	1	1	6	9
HER2				
Negative	166 (82)	167 (87)	37 (95)	30 (88)
Positive	36 (18)	26 (12)	2 (5)	4 (12)
Missing	18	24	25	25
Ki67 (%)				
Median	34	32	27	28
Range	2–89	3–88	7–53	9–51
Missing	16	14	41	43
Histopathological type				
Ductal/NST	177 (86)	167 (82)	32 (74)	33 (85)
Lobular	16 (8)	18 (9)	6 (14)	3 (8)
Medullary	10 (5)	10 (5)	4 (9)	1 (3)
Other	4 (2)	8 (4)	1 (2)	2 (5)
Missing	13	14	21	20
TILs (%)				
< 10	111 (51)	116 (54)	18 (58)	7 (35)
10–49	79 (36)	67 (31)	7 (23)	8 (40)
50–100	28 (13)	34 (16)	6 (19)	5 (25)
Missing	2	0	33	39
PAM50 subtype				
Luminal A	101 (46)	90 (42)	–	–
Luminal B	41 (19)	42 (19)	–	–
HER2-enriched	39 (18)	35 (16)	–	–
Basal-like	39 (18)	50 (23)	–	–
Missing	0	0	59	59

Patient and tumor characteristics for the whole study cohort with (*n* = 437) and without (*n* = 123) available gene expression, respectively, stratified by study arm

*ER* estrogen receptor, *HER2* human epidermal growth factor receptor 2, *NHG* Nottingham histological grade, *NST* no special type, *PR* progesterone receptor, *TAM* tamoxifen, *TILs* tumor-infiltrating lymphocytes

**Table 2:** Patient and tumor characteristics for the ER+/HER2− subgroup (*n* = 236) by treatment arm

Characteristics	ER+/HER2– cohort (*n* = 236)	
	Control group* n* (%)	Tam-treated group* n* (%)
Age (years)		
Median	46	45
Range	27–54	33–57
Tumor size (mm)		
≤ 20	56 (45)	43 (38)
> 20	68 (55)	69 (62)
Nodal status		
Node-negative	29 (23)	31 (28)
Node-positive	95 (77)	81 (72)
N1	65 (52)	58 (52)
N2	30 (24)	23 (21)
NHG		
1	20 (16)	19 (17)
2	72 (59)	61 (56)
3	31 (25)	30 (27)
Missing	1	2
PR		
Positive	117 (94)	100 (89)
Negative	7 (6)	12 (11)
Ki67 (%)		
Median	27	26
Range	2–68	5–56
Missing	8	5
Histopathological type		
Ductal/NST	105 (85)	94 (84)
Lobular	13 (11)	12 (11)
Medullary	2 (2)	1 (1)
Other	3 (2)	5 (5)
Missing	1	0
TILs (%)		
< 10	80 (65)	83 (74)
10–49	34 (27)	26 (23)
50–100	10 (8)	3 (3)
PAM50 subtype		
Luminal A	82 (66)	66 (59)
Luminal B	33 (27)	36 (32)
HER2-enriched	8 (7)	4 (4)
Basal-like	1 (1)	6 (5)

*ER* estrogen receptor, *HER2* human epidermal growth factor receptor 2, *NHG* Nottingham histological grade, *NST* no special type, *PR* progesterone receptor, *Tam* tamoxifen, *TILs* tumor-infiltrating lymphocytes

### GEX patterns and correlation analysis

**Fig. 2:**
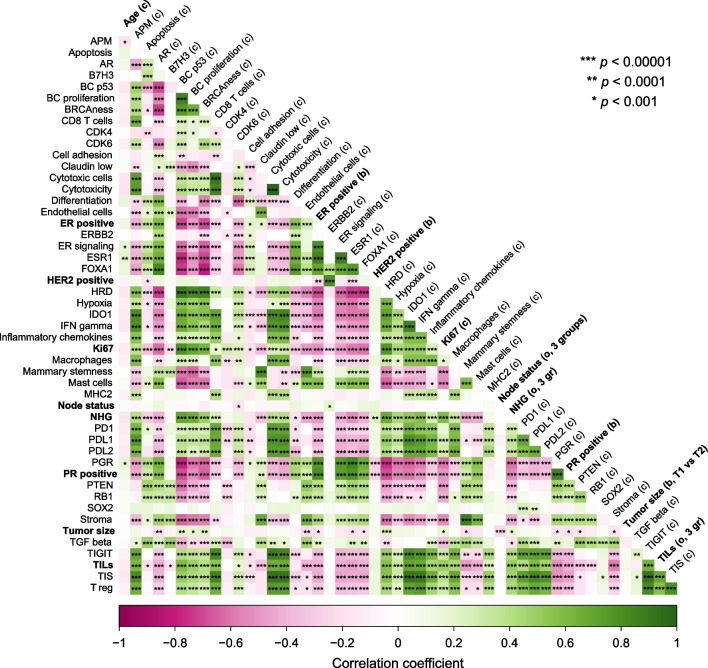
Correlation plot. Correlation between the GEX signatures and clinicopathological variables. The clinicopathological variables are indicated in bold. The labels on the diagonal contain a variable descriptor where the variables are described as continuous (c), binary (b), or ordinal (o). Significance levels represent crude *p* values. Only abbreviated GEX signature names are shown. Complete names are found in the abbreviation list. *ER* estrogen receptor, *GEX* gene expression, *HER2* human epidermal growth factor receptor 2, *NHG* Nottingham histological grade, *PR* progesterone receptor, *TILs* tumor-infiltrating lymphocytes, *T1* tumor size ≤ 20 mm, *T2* tumor size > 20 mm

As depicted in the correlation plot (Fig. 2), ESR1 was strongly correlated with the GEX signatures Mast cells and ER signaling as well as the protein levels of ER and PR. Furthermore, BC proliferation and Hypoxic GEX signatures were strongly correlated with Ki67 and NHG, and TILs were clearly associated with immune signatures. Additional file 3 illustrates that most ER-positive tumors also had higher levels of *ESR1* GEX.

**Fig. 3:**
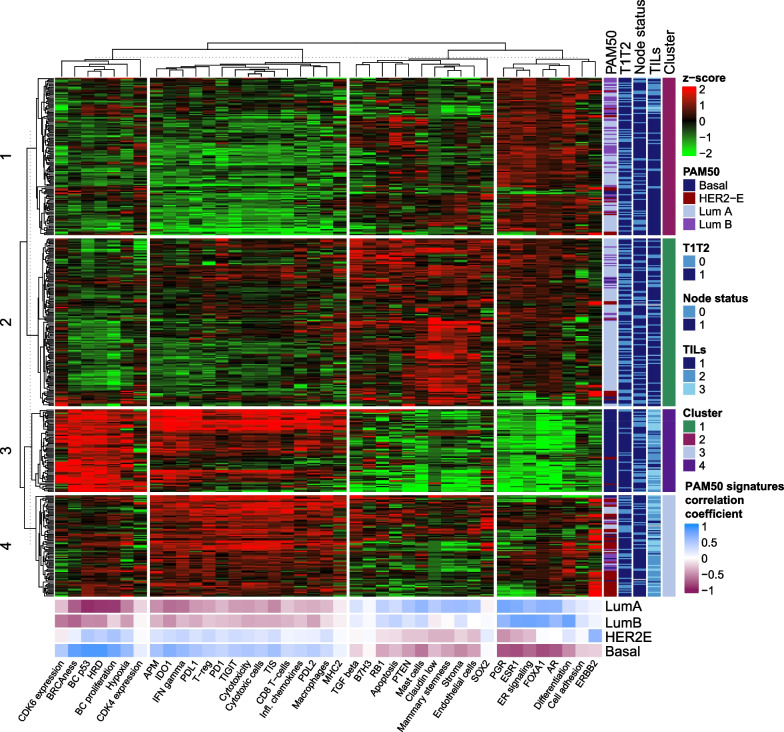
Heatmap illustrating expression levels of the GEX signatures. Heatmap of GEX signatures for all patients (*n* = 437); tumors in rows, and GEX signatures in columns. Expression levels are presented as z-scores from low (green) to high (red) expression. The panels on the right show the intrinsic subtype, tumor size, node status, and TILs score for each tumor. 1–4 illustrate the four GEX signature clusters generated using k-means clustering. The bottom panels present the relationships between PAM50 subtypes and GEX signatures, both as continuous variables and color-coded according to Pearson correlation coefficients. *GEX* gene expression, *HER2-E* human epidermal growth factor receptor 2-enriched, *Lum* luminal, *TILs* tumor-infiltrating lymphocytes, *T1* tumor size ≤ 20 mm, *T2* tumor size > 20 mm

The expression levels of BC360 GEX signatures for all 437 samples are presented in a heatmap (Fig. 3). Horizontally, four clusters with different characteristics were identified. Clusters 1 and 2 represent a hormone-receptive expression pattern similar to that of Luminal A and B tumors, where cluster 1 appears more immunoactive. In addition, the third and fourth clusters represent tumors with immunoactive GEX signatures, but cluster 3 presents lower genomic instability and high expression of ERBB2, similar to the HER2-E subtype, and the fourth cluster, which mainly includes basal-like tumors, is related to genomic instability.

### Predictive effect of GEX signatures for tamoxifen benefit in the ER+/HER2− cohort

**Fig. 4:**
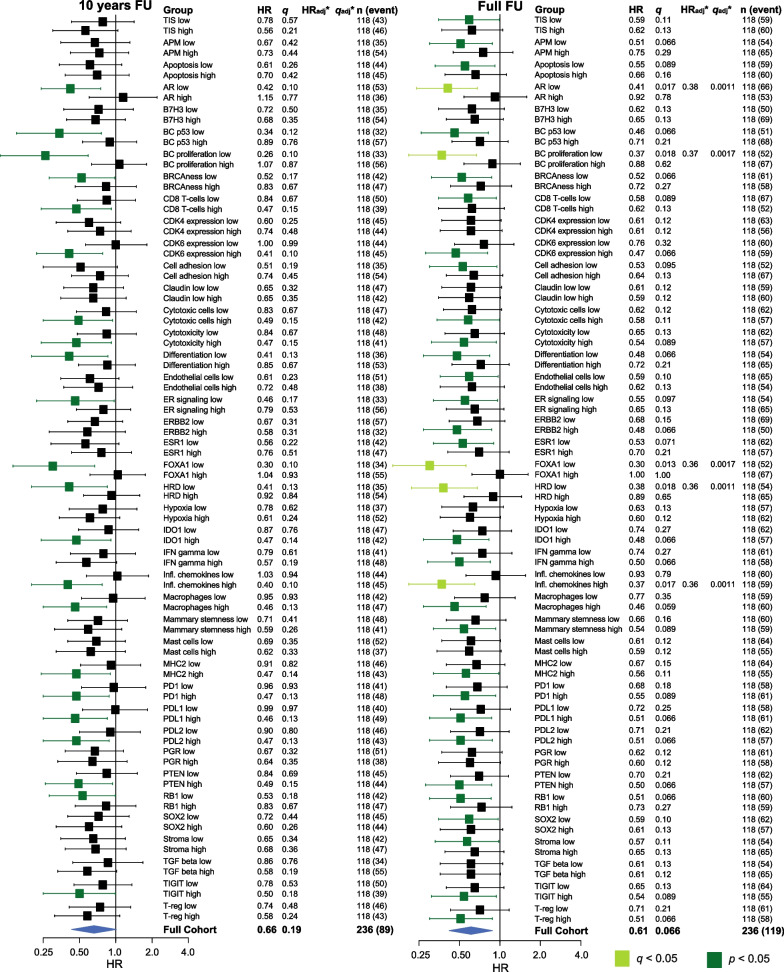
**a**, **b** Tamoxifen effect in relation to GEX signatures and RFi. Forest plots illustrating the effect of tamoxifen on RFi in patients with ER+/HER2− tumors. Plots represent results from univariable Cox regression, with HR plotted with 95% CI, and the color corresponds to the significance level. The results from the univariable Cox regression analysis are presented as HR and the corresponding *q* (FDR-adjusted *p*-value). * Results from multivariable Cox regression analyses adjusted for PAM50 subtype, node category, age, NHG, and tumor size calculated only for signatures where the univariable Cox regression *p* was < 0.05. *adj.* adjusted, *CI* confidence interval, *ER* estrogen receptor, *FDR* false discovery rate, *FU* follow-up, *GEX* gene expression, *HER2* human epidermal growth factor receptor 2, *n* number of patients, *NHG* Nottingham histological grade, *RFi* recurrence-free interval

**Fig. 5:**
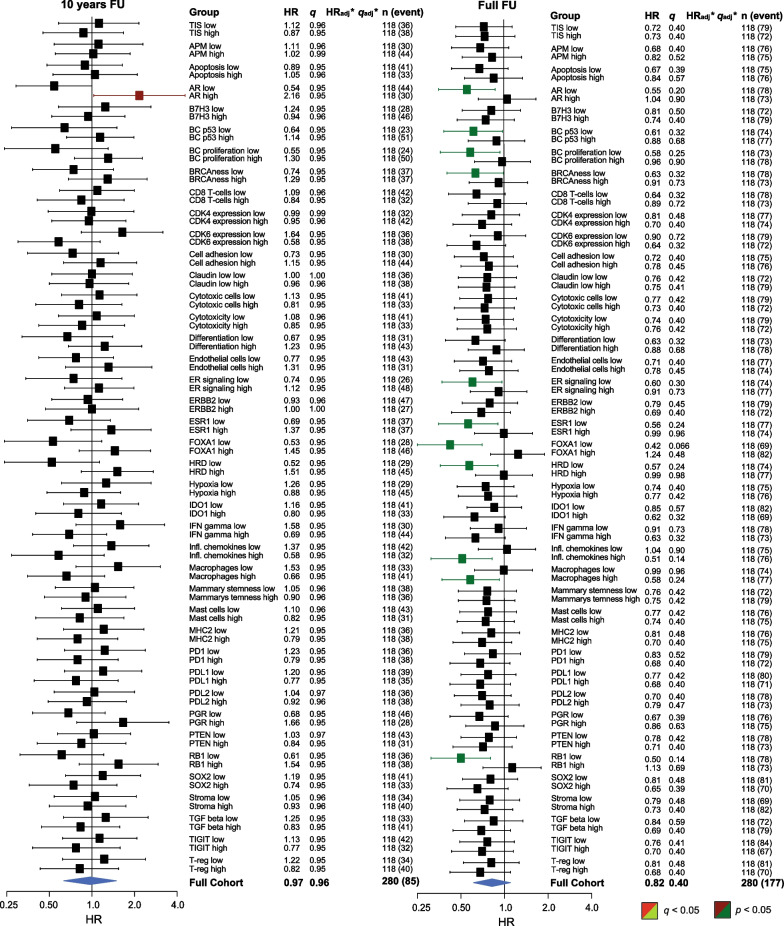
**a**, **b** Tamoxifen effect in relation to GEX signatures and OS. Forest plots illustrating the effect of tamoxifen on OS in patients with ER+/HER2− tumors. Plots represent results from univariable Cox regression analyses, with HR plotted with 95% CI, and the color corresponds to the significance level. The results from univariable Cox regression analysis are presented as HR with corresponding *q* (FDR-adjusted* p* value). * Results from multivariable Cox regressions adjusted for PAM50 subtype, node category, age, NHG, and tumor size, calculated only for signatures where the univariable Cox regression *p* was < 0.05. *adj.* adjusted, *CI* confidence interval, *ER* estrogen receptor, *FDR* false discovery rate, *FU* follow-up, *GEX* gene expression, *HER2* human epidermal growth factor receptor 2, *n* number of patients, *NHG* Nottingham histological grade, *OS* overall survival

**Fig. 6:**
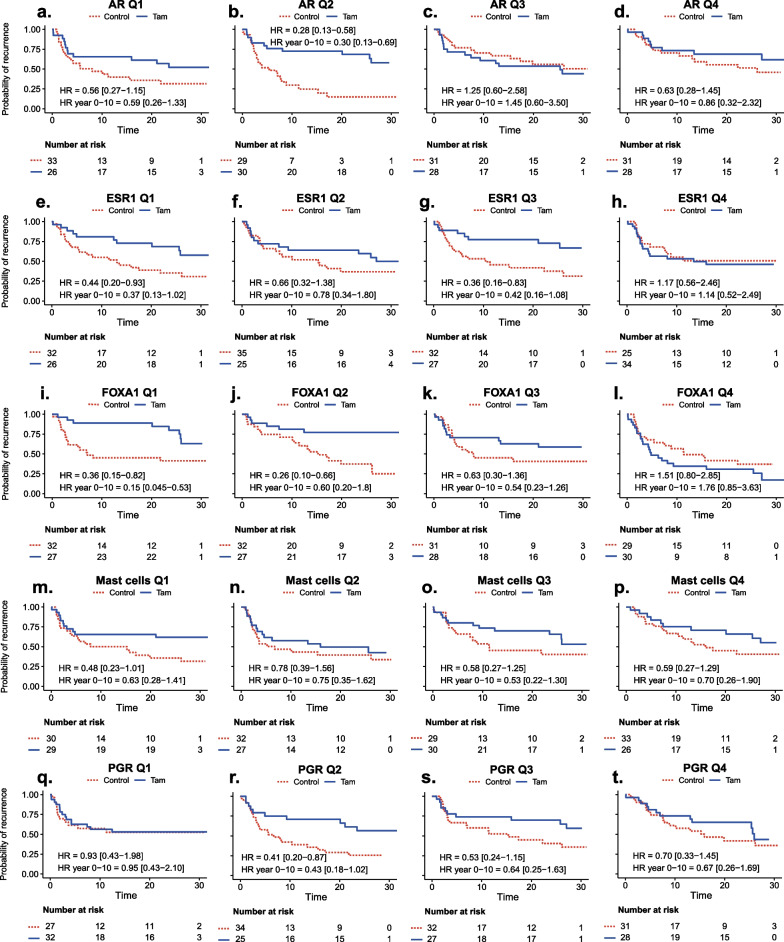
**a–t** Recurrence-free interval (RFi) and benefits of tamoxifen in GEX signature quartiles (Q1–Q4). Kaplan–Meier plots for each quartile of selected GEX signatures stratified by treatment (Tam *vs*. control) in patients with ER+/HER2− tumors; **a**–**d** *AR*, **e**–**h** *ESR1*, **i**–**l** FOXA1, **m**–**p** Mast cells and **q**–**t** *PGR*. Hazard ratios (HRs) with 95% confidence intervals are shown for the full-time follow-up and the first 10 years. *ER* estrogen receptor, *GEX* gene expression, *HER2* human epidermal growth factor receptor 2, *HR* hazard ratio, *Q* quartile, *RFi* recurrence-free interval, *Tam* tamoxifen

**Fig. 7:**
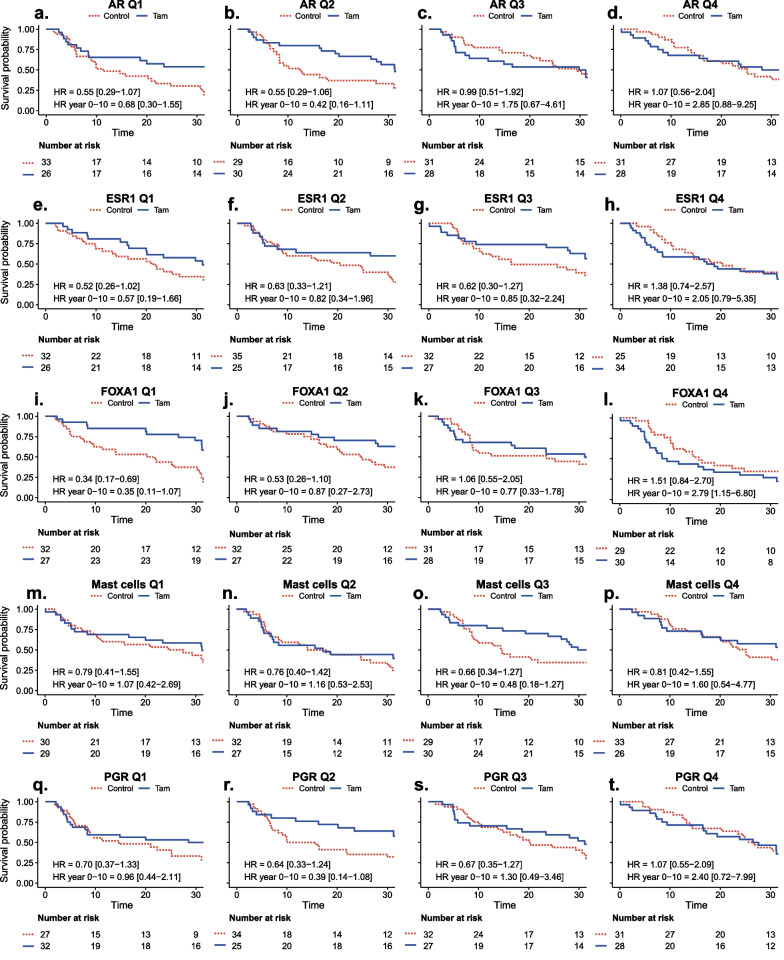
**a–t** Overall survival (OS) and benefit of tamoxifen in quartiles of GEX signatures (Q1–Q4). Kaplan–Meier plots for each quartile of selected GEX signatures stratified by treatment (Tam *vs*. control) in patients with ER+/HER2− tumors; **a**–**d** *AR*, **e**–**h** *ESR1*, **i**–**l** FOXA1, **m**–**p** Mast cells and **q**–**t** *PGR*. HRs with 95% CI are shown for the full-time follow-up and the first 10 years. *CI* confidence interval, *ER* estrogen receptor, *GEX* gene expression, *HER2* human epidermal growth factor receptor 2, *HR* hazard ratio, *OS* overall survival, *Q* quartile, *Tam* tamoxifen

Most patients in the ER+/HER2− cohort were lymph node-positive (N1), classified as Luminal A, of ductal histopathological type, PR-positive, and had low TILs levels (Table 2). The forest plots in Figs. 4 and 5 illustrate the effect of treatment (tamoxifen *vs*. control) for all GEX signatures (high and low values based on the median) for RFi (Fig. 4) and OS (Fig. 5) after 10 years and at full follow-up. The HRs were generally below 1.0, indicating that most patients with ER+/HER2− tumors did benefit from tamoxifen, which is in line with previous study results for this trial [29]. Kaplan–Meier estimates stratified by treatment for the GEX quartiles of *AR, ESR1, FOXA1*, Mast cells, and *PGR* are presented for RFi and OS in Figs. 6 and 7. Potential interactions are also illustrated in Additional files 4 and 5: Figs. S4 and S5, where the relationships between the GEX quartiles and outcome are presented in Kaplan–Meier curves for the whole ER+/HER2− part of the cohort, as well as for each treatment arm separately.

**Table 3:** Interaction terms for tamoxifen effect (ER+/HER2− cohort) for 10 years of follow-up

Gene signature	RFi				OS			
	Univariable		Multivariable^a^		Univariable		Multivariable^a^	
	HR (95% CI)	*p* (*q*)	HR (95% CI)	*p* (*q*)	HR (95% CI)	*p* (*q*)	HR (95% CI)	*p* (*q*)
AR	1.14 (0.76–1.72)	0.52 (0.87)	1.20 (0.80–1.81)	0.37 (0.62)	1.38 (0.88–2.16)	0.16 (0.20)	1.48 (0.95–2.30)	0.082 (0.10)
ESR1	1.38 (0.90–2.11)	0.15 (0.37)	1.27 (0.80–2.00)	0.31 (0.62)	1.57 (0.98–2.53)	0.062 (0.16)	1.61 (0.97–2.66)	0.066 (0.10)
FOXA1	2.24 (1.45–3.45)	0.00027 (0.0013)	2.00 (1.27–3.14)	0.0027 (0.014)	2.25 (1.42–3.56)	0.00058 (0.0029)	2.16 (1.32–3.52)	0.0021 (0.011)
Mast cells	0.97 (0.64–1.47)	0.89 (0.89)	1.03 (0.68–1.56)	0.88 (0.92)	1.00 (0.64–1.55)	0.98 (0.98)	1.04 (0.66–1.62)	0.88 (0.88)
PGR	0.97 (0.65–1.44)	0.88 (0.89)	1.02 (0.68–1.55)	0.92 (0.92)	1.40 (0.92–2.12)	0.12 (0.19)	1.54 (0.99–2.39)	0.054 (0.10)

The HR:s presented are for the multiplicative interaction term between each gene signature (unit 1 SD) and treatment (binary) in models including also the main effects for gene signature and treatment. Hence, an interaction HR of 1.00 corresponds to an effect of treatment which does not vary with expression of the gene signature, while interaction HR ≠ 1.00 suggests that an increase in the gene signature score associates with tamoxifen treatment being less effective or more effective in preventing the event of interest, for interaction HR > 1.00 and HR < 1.00 respectively

Interaction terms for the ER+/HER2− cohort of tamoxifen treatment and selected gene signatures as continuous scores, estimated by Cox proportional hazards regression

^a^Adjusted for PAM50 subtype, node status, NHG, age, and tumor size

*CI* confidence interval, *ER* estrogen receptor, *HER2* human epidermal growth factor receptor 2, *HR* hazard ratio, *OS* overall survival, *RFi* recurrence-free interval, *SD* standard deviation

**Table 4:** Interaction terms for tamoxifen effect (ER+/HER2− cohort) for full follow-up

Gene signature	RFi				OS			
	Univariable		Multivariable^a^		Univariable		Multivariable^a^	
	HR (95% CI)	*p* (*q*)	HR (95% CI)	*p* (*q*)	HR (95% CI)	*p* (*q*)	HR (95% CI)	*p* (*q*)
AR	1.11 (0.77–1.58)	0.59 (0.78)	1.14 (0.80–1.64)	0.47 (0.67)	1.26 (0.91–1.76)	0.17 (0.28)	1.33 (0.96–1.86)	0.090 (0.15)
ESR1	1.36 (0.93–1.98)	0.12 (0.30)	1.26 (0.85–1.89)	0.25 (0.63)	1.50 (1.07–2.11)	0.020 (0.050)	1.40 (0.98–1.99)	0.063 (0.15)
FOXA1	1.87 (1.28–2.75)	0.0013 (0.0064)	1.61 (1.08–2.38)	0.018 (0.091)	1.89 (1.34–2.67)	0.00032 (0.0016)	1.72 (1.21–2.46)	0.0027 (0.014)
Mast cells	1.09 (0.76–1.56)	0.66 (0.78)	1.12 (0.78–1.60)	0.54 (0.67)	1.00 (0.74–1.37)	0.98 (0.98)	1.04 (0.76–1.41)	0.83 (0.83)
PGR	0.95 (0.66–1.36)	0.78 (0.78)	1.00 (0.68–1.45)	0.98 (0.98)	1.18 (0.85–1.62)	0.32 (0.40)	1.22 (0.88–1.69)	0.24 (0.30)

The HR:s presented are for the multiplicative interaction term between each gene signature (unit 1 SD) and treatment (binary) in models including also the main effects for gene signature and treatment. Hence, an interaction HR of 1.00 corresponds to an effect of treatment which does not vary with expression of the gene signature, while interaction HR ≠ 1.00 suggests that an increase in the gene signature score associates with tamoxifen treatment being less effective or more effective in preventing the event of interest, for interaction HR > 1.00 and HR < 1.00 respectively

Interaction terms for the ER+/HER2− cohort of tamoxifen treatment and selected gene signatures as continuous scores, estimated by Cox proportional hazards regression

*CI* confidence interval, *ER* estrogen receptor; HER2, human epidermal growth factor receptor 2; HR, hazard ratio; OS, overall survival; RFi, recurrence-free interval; SD, standard deviation

^a^Adjusted for PAM50 subtype, node status, NHG, age, and tumor size

With respect to RFi, high *AR* expression was associated with worse outcomes following tamoxifen treatment after 10 years of follow-up (HR_*AR*(high)_ = 1.15, 95% CI = 0.60–2.20, *q* = 0.77; HR_*AR*(low)_ = 0.42, 95% CI = 0.24–0.75, *q* = 0.10) (Fig. 4), corresponding to a significant interaction effect between dichotomized *AR* expression and tamoxifen treatment (*p*_interaction_ = 0.02). However, the evidence for an interaction was much weaker (*p*_interaction_ = 0.52, Tables 3, 4) when *AR* was analyzed as a continuous variable, indicating no clear dose–response relationship. Similar results were observed for full-time follow-up (Fig. 4) and OS (Fig. 5). This pattern can also be observed in Figs. 6 and 7a–d, in which the effect of tamoxifen was assessed in the quartiles of *AR* expression.

There was a trend toward a better tamoxifen effect for those defined as *ESR1* low compared to high (HR_RFi *ESR1*(high)_ = 0.76, 95% CI = 0.43–1.35, *q* = 0.51; HR_RFi *ESR1*(low)_ = 0.56_,_ 95% CI = 0.29–1.06, *q* = 0.22), which was more pronounced with full-time follow-up (Fig. 4). Similar results were observed for OS (Fig. 5). The strongest evidence for *ESR1* × treatment interaction was observed in OS after full-time follow-up (*p*_interaction_ = 0.02, Tables 3, 4). As shown in Figs. 6 and 7e–h, the results were similar for the GEX quartiles of *ESR1*.

A similar trend was observed for *FOXA1*, indicating that low expression was associated with an improved tamoxifen benefit for 10 years RFi (HR_RFi *FOXA1*(high)_ = 1.04, 95% CI = 0.61–1.76, *q* = 0.93; HR_RFi *FOXA1*(low)_ = 0.30, 95% CI = 0.14–0.67, *q* = 0.10, Figs. 4 and 6i–l) and after full-time follow-up and OS (Figs. 5 and 7i–l). The interaction between *FOXA1* GEX and tamoxifen treatment was significant for RFi after 10 years of follow-up in univariable (*p* < 0.001) and multivariable analyses adjusted for other clinicopathological factors (*p* = 0.003). Similar results were obtained for the full-time follow-up and OS (Tables 3, 4). After adjusting for FDR, all *FOXA1* × treatment interactions remained statistically significant, except for the multivariable regression for RFi after full follow-up (Tables 3, 4).

Another way of illustrating potential interactions between tamoxifen treatment and *FOXA1*, *AR*, *ESR1*, and *PGR* expression is shown in Additional files 4 and 5: Figs. S4 and S5, where Kaplan–Meier estimates are presented in the tamoxifen and control arms separately in relation to RFi (Additional file 4: Fig. S4) and OS (Additional file 5: Fig. S5). In line with the above-presented predictive analyses, increasing *FOXA1* quartiles show a strong association to worse prognosis in relation to both endpoints in patients with ER+/HER2− tumors allocated to adjuvant tamoxifen, but not in the ER+/HER2− control group (Additional files 4 and 5: Figs. S4 and S5, g–i). For *AR*, a trend is observed that lower expression is related to worse outcome for both endpoints in the untreated group, but not in the tamoxifen group (Additional files 4 and 5: Figs. S4 and S5, a–c). For *ESR1*, the highest expression quartile appears to be related to poor outcome only in the tamoxifen treated group for both endpoints (Additional files 4 and 5: Figs. S4 and S5, d–f).

No clear difference in the effect of tamoxifen was demonstrated in relation to the Mast cell signature or *PGR,* indicating a similar tamoxifen benefit regardless of the GEX level of these signatures (Figs. 4, 5 and 6, 7m–t, and Tables 3, 4). For RFi, there were trends of improved tamoxifen effects in relation to several GEX signatures, including the low GEX of the tumor mutational response signatures, BC p53, BRCAness, and HRD. Similar results were noted for the tumor regulation signatures differentiation and *RB1*, high GEX of *CDK6,* and *PTEN*, and signatures related to tumor immune activity and inhibitory immune signaling.

### Prognostic effect of GEX signatures in the whole cohort, regardless of IHC subtype

**Fig. 8:**
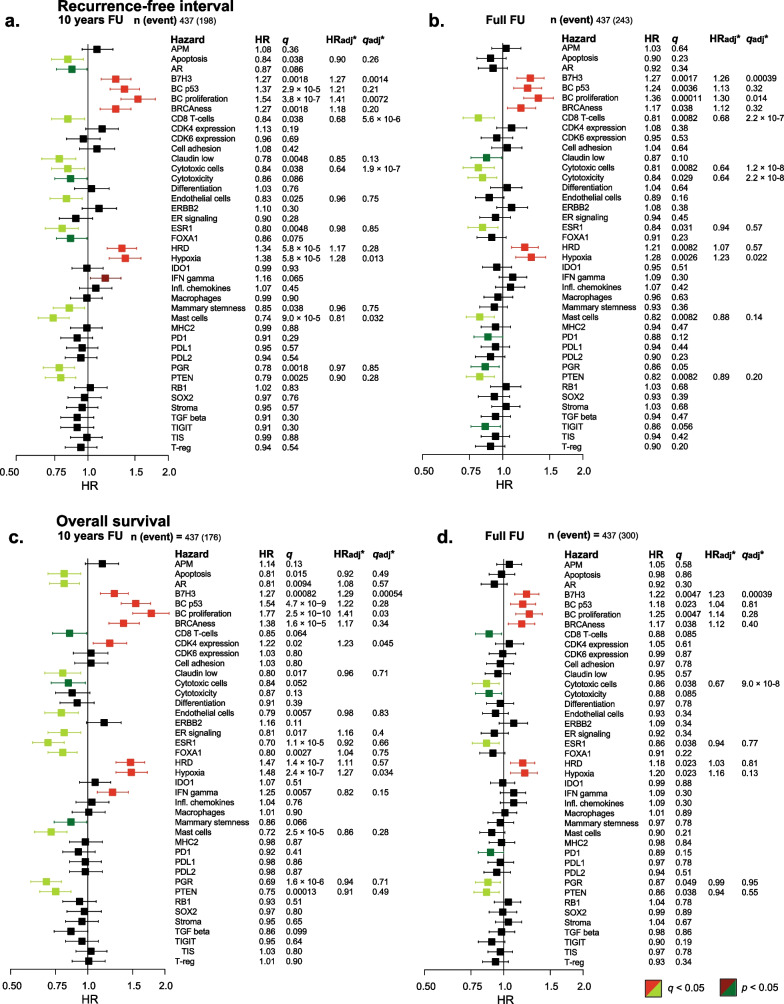
**a–d** Forest plot of GEX signatures and association to outcomes. Outcomes of GEX signatures as continuous variables in all patients for **a** RFi at 10 years of follow-up, **b** RFi at full follow-up, **c** OS at 10 years, and **d** OS at full follow-up. All plots **a**–**d** represent data from the entire cohort for which GEX data were available (*n* = 437). Plots represent data from univariable Cox regression, with HR plotted with 95% CI, and the color corresponds to the significance level. Data from univariable Cox regressions are presented as HR with the corresponding *q* (FDR-adjusted *p*-value). *Data from multivariable Cox regressions adjusted for PAM50 subtype, node category, age, NHG, tumor size, and tamoxifen arm, calculated only for signatures where the univariable Cox regression *p* was < 0.05. *adj* adjusted, *CI* confidence interval, *ER* estrogen receptor, *FDR* false discovery rate, *FU* follow-up, *GEX* gene expression, *HR* hazard ratio, *HER2* human epidermal growth factor receptor 2, *n* number of patients, *NHG* Nottingham histological grade, *OS* overall survival, *RFi* recurrence-free interval

**Fig. 9:**
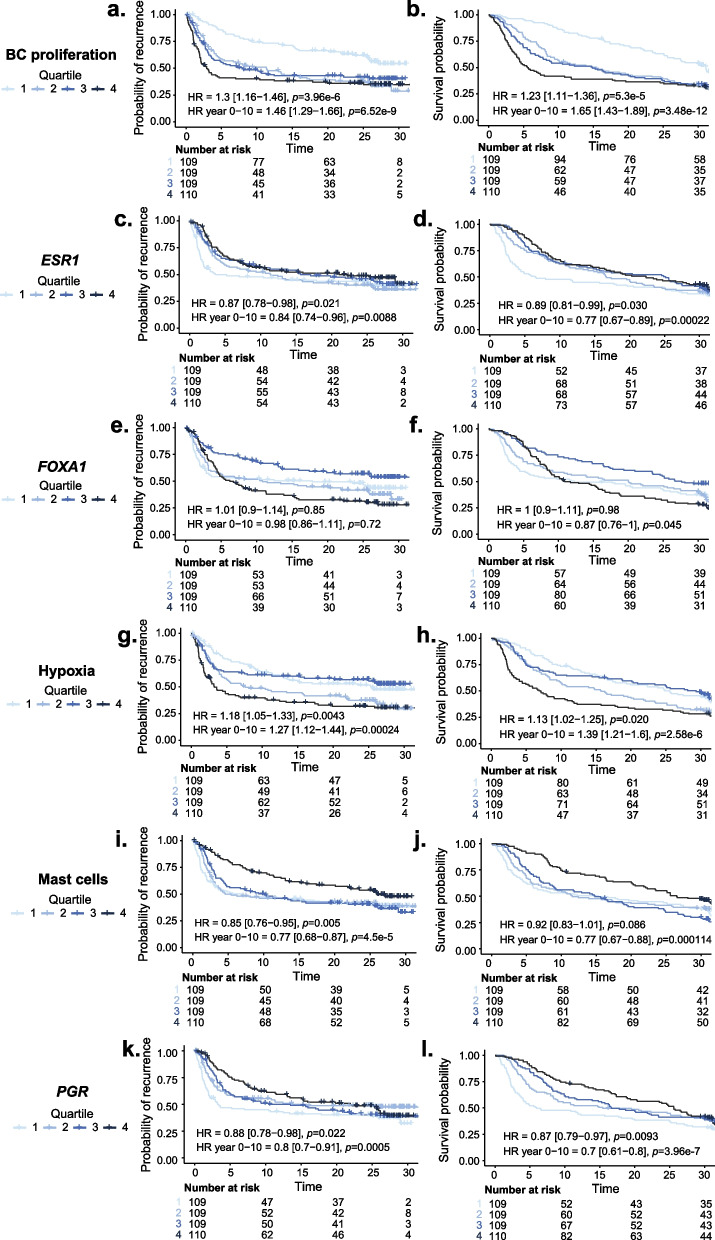
**a–l** Outcomes (for RFi and OS) of GEX signatures. Kaplan–Meier plots representing the relationship between RFi or OS and GEX levels in terms of quartiles (Q1–Q4) for the whole cohort (*n* = 437) for **a**–**b** BC proliferation, **c**–**d** *ESR1*, **e**–**f** *FOXA1*, **g**–**h** hypoxia, **i**–**j** Mast cells, and **k–l** *PGR*. HRs with 95% CI are shown for the full-time follow-up and the first 10 years. *CI* confidence interval, *ER* estrogen receptor, *GEX* gene expression, *HER2* human epidermal growth factor receptor 2, *HR* hazard ratio, *OS* overall survival, *Q* quartile, *RFi* recurrence-free interval, *Tam* tamoxifen

The associations between the BC360 assay GEX signatures as continuous scores and outcomes (RFi and OS), analyzed in the full cohort, are presented in Fig. 8a–d. Kaplan–Meier curves for these outcomes are illustrated in Fig. 9 for the quartiles of the selected GEX signatures: BC proliferation, *ESR1*, *FOXA1*, Hypoxia, Mast cells, and *PGR*.

After 10 years of follow-up, higher expression of *AR, ESR1*, *PGR* and the Mast cells signature was associated with better outcomes in terms of RFi (Fig. 8a–b, HR_*AR*_ = 0.87, 95% CI = 0.76–0.99, *q* = 0.086, HR_*ESR1*_ = 0.80, 95% CI = 0.69–0.92,* q* = 0.005, HR_Mast cells_ = 0.74, 95% CI = 0.65–0.85, *q* < 0.0001, and HR_*PGR*_ = 0.78, 95% CI = 0.68–0.89, *q* = 0.002). This was also true for OS (Fig. 8c–d). As illustrated in Fig. 9, the prognostic effects of these signatures were more prominent with increased expression level. A decreased RFi was also noted for high *FOXA1* GEX levels (HR_*FOXA1*_ = 0.86, 95% CI = 0.76–0.99, *q* = 0.075); however, no clear dose–response relationship was observed (Fig. 9). In contrast to the above results, an increased RFi after the same follow-up period was linked to higher expression of the BC proliferation (HR_BC proliferation_ = 1.54, 95% CI = 1.33–1.79, *q* < 0.0001) and Hypoxia (HR_Hypoxia_ = 1.38, 95% CI = 1.20–1.58, *q* < 0.0001) signatures. The results were also significant after adjusting for other clinicopathological factors (all *q* < 0.05).

Another signature worth noting is B7-H3, which seemed to be an independent unfavorable prognostic marker in relation to RFi after 10 years of follow-up (HR_B7-H3_ = 1.27, 95% CI = 1.12–1.45, *q* = 0.002) as well as OS (HR_B7-H3_ = 1.27, 95% CI = 1.12–1.44, *q* = 0.0008). In contrast, within the first 10 years of follow-up, the Claudin-low signature was associated with better outcomes in terms of both RFi (HR_Claudin low_ = 0.78, 95% CI = 0.67–0.90, *q* = 0.005) and OS (HR_Claudin low_ = 0.80, 95% CI = 0.68–0.94, *q* = 0.02). Other signatures of prognostic value, even after adjusting for other clinicopathological factors, encompassed prognostically favorable and unfavorable signatures related to cytotoxic cells and signatures related to genetic tumor mutational responses (p53, BRCAness, and HRD), as well as *PTEN,* respectively. The four GEX clusters generated by the k-means clustering (Fig. 3) had prognostic value both after 10 years and at full follow-up (Additional file 6, a–b). However, PAM50 provided a higher prognostic value than these clusters (Additional file 6, c–b).

## Discussion

In the present study, the predictive value of GEX signatures for tamoxifen effect in premenopausal breast cancer patients with early ER+/HER2− tumors was explored. We observed associations between low expression of *AR*, *FOXA1,* and surprisingly, *ESR1* and improved benefit of tamoxifen. Moreover, in the whole cohort, we found a prognostic effect for each of the GEX signatures BC proliferation, Hypoxia, Mast cells, and the GEX of *AR*, *ESR1*, and *PGR,* even after adjustment for established prognostic factors.

We have previously demonstrated that two years of adjuvant tamoxifen is effective for long-term breast cancer-related survival for patients with ER+ tumors from this trial [29], and that the effect of adjuvant tamoxifen therapy only seemed beneficial in patients with Luminal A tumors, as assessed by PAM50 [9]. *ESR1* GEX positively correlated with ER and PR protein levels and the Luminal A subtype. Furthermore, high expression of the BC proliferation and Hypoxia GEX signatures was strongly correlated with high Ki67, high NHG and a Basal-like subtype. This was also reflected in the prognostic analyses, in which these signatures were associated with poor outcomes.

All selected 41 GEX signatures were included in exploratory predictive analyses. The GEX of *AR* is known to be associated with luminal subtypes and better outcomes [18, 40], and a similar prognostic effect of *AR* was noted in our study. Interestingly, our results indicate that a high *AR* GEX level is associated with a negative effect of tamoxifen after ten years, for both RFi and OS. However, no significant *AR*-by-treatment interactions were observed. Previous preclinical data suggest that *AR* overexpression might induce tamoxifen resistance; therefore, additional treatments such as AR inhibition may benefit these patients [19, 41]. However, data from clinical trials including patients with ER+ tumors that support the use of AR inhibitors are sparse. Additionally, the results of the study are expected to be influenced by the selection of patients with ER+ and HER2− tumors. However, the selection of patients with a defined phenotype makes clinical interpretation more relevant by reducing tumor heterogeneity in the cohort in which GEX signatures are evaluated.

In line with previous studies, we found that patients with high *ESR1* GEX had better outcome [17]. Since ER protein expression is associated with a better response to endocrine therapy, and *ESR1* GEX is positively correlated with ER status, an expression-dependent relationship between *ESR1* expression and tamoxifen benefits [42] may be anticipated. In contrast to our results, a high *ESR1* expression was a strong predictor of tamoxifen benefits in ER+ breast cancer in the National Surgical Adjuvant Breast and Bowel Project (NSABP) B-14 trial [16]. Kuske et al. stated that endocrine resistance to aromatase inhibitors can be linked to high ER expression and reduced ER phosphorylation [43], and other mechanisms of ER resistance have been proposed based on results in the metastatic setting, including mutations in *ESR1* [44]. Although we observed *PGR* as strong prognostic factor in this cohort, no predictive effect of tamoxifen was found, as reported in the NSABP B-14 study [16].

FOXA1 plays a critical role in the regulation of ER function and may contribute to endocrine resistance in breast cancer [45–47]. Clinically, FOXA1 protein expression has been associated with a luminal phenotype, including increased hormone receptor expression and improved outcomes [25, 26, 48, 49]. One study indicated that FOXA1 IHC staining decreased after neoadjuvant endocrine treatment, but the staining intensity (%) was not linked to treatment benefits [50]. To the best of our knowledge, no clear clinical evidence has been provided regarding the predictive effect of *FOXA1* GEX in breast cancer. In this study, we showed that the benefit of tamoxifen decreased with increasing GEX of *FOXA1*, revealing a group of patients with ER+/HER2− tumors and low expression of *FOXA1* who had an excellent response to tamoxifen treatment. In line with our results, previous studies have suggested that overexpression and mutation of *FOXA1* could be underlying factors in endocrine resistance [46, 51]. In contrast to the observation that high *FOXA1* reduces the benefit of tamoxifen in the ER+/HER2− subgroup, we observed high *FOXA1* GEX to be prognostically favorable in the whole cohort, although no clear dose–response relationship was observed. A possible explanation for this may be the association between FOXA1 expression and luminal traits. In a subgroup analysis including only ER+/HER2− tumors, to mitigate this possible confounder, high *FOXA1* GEX was a negative prognostic factor for both RFi (Additional file 4 g) and OS (Additional file 5 g). Interestingly, high *FOXA1* was strongly associated with inferior outcome in the ER+/HER2− subgroup of patients allocated to tamoxifen, which was not true for the corresponding patients in the control arm. Together, these results strongly support that *FOXA1* is a putative tamoxifen-predictive factor in patients with ER+/HER2− tumors.

Previously, we reported PAM50 subtypes to have prognostic relevance in this premenopausal cohort [9]. Although we identified four GEX clusters with prognostic effects in this cohort, these did not outperform PAM50 (Additional file 6). Focusing on the respective GEX signatures of BC360, those related to proliferation, hypoxia, immunology, and hormone receptors were associated with long-term prognosis in this cohort. High expression of BC proliferation and hypoxia gene signatures was associated with worse RFi and OS outcomes. An association between BC proliferation and poor outcome was expected, because *MKI67*, which encodes Ki67, is included in this signature. Ingebriktsen et al. demonstrated that a 6 Gene Proliferation Score (6GPS) incorporating proliferation in young breast cancer patients (< 40 years) is of prognostic significance [21]. Oncotype DX includes 5 of the 16 genes of the BC proliferation GEX signature, further illustrating how proliferation markers at the RNA level can be of clinical interest [22]. Several research groups have also shown that hypoxia-related GEX profiles have prognostic value in breast cancer, which supports our results [23, 52, 53].

We have previously shown that TILs are independently associated with prognosis in premenopausal patients [27]. Mast cells are a part of the innate immune system and are more frequent in hormone receptor-positive breast cancers [54]. The Mast cell GEX signature incorporated multiple genes (Additional file 2), and we demonstrated a possible association between high expression of this signature and better prognosis. Another Mast cell gene signature (MCS) has been shown to be prognostic and suggested as a potential indicator of immunotherapy response for patients with head and neck squamous cell carcinoma [55]. In early TNBC, the benefit from capecitabine has been demonstrated to be linked to the Mast cell signature used in our study [15]. Data on the endocrine therapy-predictive effects of this signature in early breast cancer are lacking, and predictive effects were not observed in our cohort.

The strengths of this study include its pure premenopausal cohort, long-term follow-up, and randomized design. Furthermore, the tumor material in this cohort was treatment-naïve, making the GEX readings representative of newly diagnosed tumors. We illustrated the predictive results in terms of quartiles to visualize any dose–response relationship with tamoxifen. However, the cutoffs of GEX signatures have not been settled for clinical use, and more data are needed to further explore this. The limitations of this study are the limited cohort size and, hence, low power, especially for the detection of interaction effects. Moreover, the treatment of this cohort today would differ in terms of systemic therapy from the guidelines of that time. A data-driven selection of signatures was used for some analyses, which increased the risk of false positives. However, we prespecified the evaluation of biologically important signatures such as *ESR1* and *PGR*, and the analyses were adjusted for multiple testing. Regarding the endpoints, we chose RFi rather than the breast cancer-free interval (BCFi). The difference lies in the inclusion of contralateral breast cancer (CBC; invasive and/or in situ) in the latter definition. The inclusion of the CBC would have resulted in more events; however, as in other randomized studies, including those evaluating the clinical utility of GEX assays, the CBC is often considered a censoring event. In addition, we focused on the potential effect of tamoxifen in reducing breast cancer recurrence, not as chemoprevention.

## Conclusions

In summary, this study showed an association between low gene expression of *FOXA1* and tamoxifen benefit in premenopausal patients with ER+/HER2− tumors. In addition, the findings confirmed that BC proliferation and Hypoxia gene expression signatures identify patients with a dismal prognosis. The gene expression of *ESR1*, *PGR,* and the Mast cells gene expression signature were observed to be associated with improved outcomes. The results warrant future validation in independent cohort studies.

### Supplementary Information

**Additional file 1.** Abbreviated translated study protocol

**Additional file 2**. Genes included in the gene expression signatures

**Additional file 3**. Correlation between ESR1 gene expression and ER status, based on immunohistochemistry. Abbreviations: ER, estrogen receptor, HER2, human epidermal growth factor receptor 2

**Additional file 4**. RFi in relation to quartiles of selected GEX signatures, ER+/HER2− tumors. Kaplan–Meier plots representing the relationship between RFi and GEX levels in terms of quartiles (Q1–Q4) for a–c) AR, d–f) ESR1, g–i) FOXA1, and j–l) PGR in patients with ER+/HER2− tumors (*n* = 236, left column), ER+/HER2− tumors in the control group (n = 124, middle column), and ER+/HER2− tumors treated with tamoxifen (*n* = 112, right column). Abbreviations: HER2, human epidermal growth factor receptor 2; HR, hazard ratio; ER, estrogen receptor; RFi, recurrence-free interval

**Additional file 5**. OS in relation to quartiles of selected GEX signatures, ER+/HER2− tumors. Kaplan–Meier plots representing the relationship between OS and GEX levels in terms of quartiles (Q1–Q4) for a–c) AR, d–f) ESR1, g–i) FOXA1, and j–l) PGR in patients with ER+/HER2− tumors (n = 236, left column), ER+/HER2− tumors in the control group (*n* = 124, middle column), and ER+/HER2− tumors treated with tamoxifen (*n* = 112, right column). Abbreviations: HER2, human epidermal growth factor receptor 2; HR, hazard ratio; ER, estrogen receptor; OS, overall survival

**Additional file 6**. Outcomes (RFi and OS) of the signature clusters (a–b) and PAM50 subtypes (c–d). Kaplan–Meier plots representing the relationship between four signature clusters (1–4) for the whole cohort (*n* = 437) for a) RFi and b) OS. Abbreviations: FU, follow-up; HR, hazard ratio; OS, overall survival; RFi, recurrence-free interval

## Data Availability

The datasets used and/or analyzed during the current study are available from the corresponding author upon reasonable request if this is in line with current laws.

## Abbreviations

APM: Antigen processing machinery
AR: Androgen receptor
BC: Breast cancer
BCFi: Breast cancer-free interval
BC360 panel: NanoString Breast Cancer 360™ panel
BRCAness: Breast cancer gene-ness
B7-H3: B7 homolog 3 protein
BC p53: Breast cancer tumor protein p53
CBC: Contralateral breast cancer
CDK4: Cyclin-dependent kinase 4
CDK6: Cyclin-dependent kinase 6
CD8 T-cell: A cluster of differentiation 8 T (thymus)-cell
CI: Confidence interval
DATECAN: Definition for the assessment of time-to-event endpoints in cancer trials
ER: Estrogen receptor
ERBB2: Erb-b2 receptor tyrosine kinase 2
ESR1: Estrogen receptor 1
FFPE: Formalin-fixed paraffin-embedded
FDR: False discovery rate
FOXA1: Forkhead box A1
FU: Follow-up
GEX: Gene expression
HER2: Human epidermal growth factor receptor 2
HER2-E: Human epidermal growth factor receptor 2-enriched
HIF-1α: Hypoxia-inducible factor 1α
HR: Hazard ratio
HRD: Homologous recombination deficiency
IDO1: Indoleamine 2, 3-dioxygenase 1
IFN Gamma: Interferon gamma
IHC: Immunohistochemistry
KM: Kaplan–Meier
Lum: Luminal
NHG: Nottingham histological grade
NSABP: National Surgical Adjuvant Breast, and Bowel Project
MHC2: Major histocompatibility complex 2
OS: Overall survival
PAM50: Prediction analysis of microarray 50
PD1: Programmed cell death 1
PDL-1: Programmed cell death ligand 1
PDL-2: Programmed cell death ligand 2
PGR: Progesterone receptor
PR: Progesterone receptor
PTEN: Phosphatase and tensin homolog
Q: Quartile
Rb1: Retinoblastoma 1
REMARK: Reporting Recommendations for Tumor Marker Prognostic Studies
RFi: Recurrence-free interval
RNA: Ribonucleic acid
ROR: Risk of recurrence
SD: Standard deviation
SOX2: Sex-determining region Y box transcription factor 2
sTIL: Stromal tumor-infiltrating lymphocytes (sTILs, denoted TILs in manuscript)
Tam: Tamoxifen
TGF-Beta: Transforming growth factor-beta
tft: Test for trend
TIGIT: T cell immunoreceptor with immunoglobulin and ITIM domains
TILS: Tumor-infiltrating lymphocytes
TIS: Tumor inflammation signature
TNBC: Triple-negative breast cancer
Treg: Regulatory T (thymus) cell
6GPS: 6 Gene Proliferation Score

## References

[1] Pan H, Gray R, Braybrooke J, Davies C, Taylor C, McGale P (2017). 20-Year risks of breast-cancer recurrence after stopping endocrine therapy at 5 years. N Engl J Med.

[2] Fisher B, Costantino JP, Wickerham DL, Redmond CK, Kavanah M, Cronin WM (1998). Tamoxifen for prevention of breast cancer: report of the national surgical adjuvant breast and bowel project P-1 study. J Natl Cancer Inst.

[3] Yao J, Deng K, Huang J, Zeng R, Zuo J (2020). Progress in the understanding of the mechanism of tamoxifen resistance in breast cancer. Front Pharmacol.

[4] The Early Breast Cancer Trialists' Collaborative Group (2005). Effects of chemotherapy and hormonal therapy for early breast cancer on recurrence and 15-year survival: an overview of the randomised trials. Lancet.

[5] Dunnwald LK, Rossing MA, Li CI (2007). Hormone receptor status, tumor characteristics, and prognosis: a prospective cohort of breast cancer patients. Breast Cancer Res.

[6] The Early Breast Cancer Trialists' Collaborative Group (2011). Relevance of breast cancer hormone receptors and other factors to the efficacy of adjuvant tamoxifen: patient-level meta-analysis of randomised trials. Lancet.

[7] Dowsett M, Sestak I, Lopez-Knowles E, Sidhu K, Dunbier AK, Cowens JW (2013). Comparison of PAM50 risk of recurrence score with oncotype DX and IHC4 for predicting risk of distant recurrence after endocrine therapy. J Clin Oncol.

[8] Pu M, Messer K, Davies SR, Vickery TL, Pittman E, Parker BA (2020). Research-based PAM50 signature and long-term breast cancer survival. Breast Cancer Res Treat.

[9] Lundgren C, Bendahl PO, Church SE, Ekholm M, Fernö M, Forsare C (2022). PAM50 subtyping and ROR score add long-term prognostic information in premenopausal breast cancer patients. NPJ Breast Cancer.

[10] Sparano JA, Gray RJ, Ravdin PM, Makower DF, Pritchard KI, Albain KS (2019). Clinical and genomic risk to guide the use of adjuvant therapy for breast cancer. N Engl J Med.

[11] Kalinsky K, Barlow WE, Gralow JR, Meric-Bernstam F, Albain KS, Hayes DF (2021). 21-Gene assay to inform chemotherapy benefit in node-positive breast cancer. N Engl J Med.

[12] Sestak I, Cuzick J, Dowsett M, Lopez-Knowles E, Filipits M, Dubsky P (2014). Prediction of late distant recurrence after 5 years of endocrine treatment: a combined analysis of patients from the Austrian breast and colorectal cancer study group 8 and arimidex, tamoxifen alone or in combination randomized trials using the PAM50 risk of recurrence score. J Clin Oncol.

[13] Sparano JA, Gray RJ, Makower DF, Pritchard KI, Albain KS, Hayes DF (2018). Adjuvant chemotherapy guided by a 21-gene expression assay in breast cancer. N Engl J Med.

[14] Swain SM, Tang G, Brauer HA, Goerlitz DS, Lucas PC, Robidoux A (2020). NSABP B-41, a randomized neoadjuvant trial: genes and signatures associated with pathologic complete response. Clin Cancer Res.

[15] Asleh K, Brauer HA, Sullivan A, Lauttia S, Lindman H, Nielsen TO (2020). Predictive biomarkers for adjuvant capecitabine benefit in early-stage triple-negative breast cancer in the FinXX clinical trial. Clin Cancer Res.

[16] Kim C, Tang G, Pogue-Geile KL, Costantino JP, Baehner FL, Baker J (2011). Estrogen receptor (ESR1) mRNA expression and benefit from tamoxifen in the treatment and prevention of estrogen receptor–positive breast cancer. J Clin Oncol.

[17] Tomita S, Zhang Z, Nakano M, Ibusuki M, Kawazoe T, Yamamoto Y (2009). Estrogen receptor α gene ESR1 amplification may predict endocrine therapy responsiveness in breast cancer patients. Cancer Sci.

[18] Vidula N, Yau C, Wolf D, Rugo HS (2019). Androgen receptor gene expression in primary breast cancer. NPJ Breast Cancer.

[19] De Amicis F, Thirugnansampanthan J, Cui Y, Selever J, Beyer A, Parra I (2010). Androgen receptor overexpression induces tamoxifen resistance in human breast cancer cells. Breast cancer Res Treat.

[20] Kensler KH, Regan MM, Heng YJ, Baker GM, Pyle ME, Schnitt SJ (2019). Prognostic and predictive value of androgen receptor expression in postmenopausal women with Estrogen receptor-positive breast cancer: results from the Breast International Group Trial 1–98. Breast Cancer Res.

[21] Ingebriktsen LM, Finne K, Akslen LA, Wik E (2022). A novel age-related gene expression signature associates with proliferation and disease progression in breast cancer. Br J Cancer.

[22] Paik S, Shak S, Tang G, Kim C, Baker J, Cronin M (2004). A multigene assay to predict recurrence of tamoxifen-treated, node-negative breast cancer. N Engl J Med.

[23] Tutzauer J, Sjöström M, Holmberg E, Karlsson P, Killander F, Leeb-Lundberg LMF (2022). Breast cancer hypoxia in relation to prognosis and benefit from radiotherapy after breast-conserving surgery in a large, randomised trial with long-term follow-up. Br J Cancer.

[24] Wallden B, Storhoff J, Nielsen T, Dowidar N, Schaper C, Ferree S (2015). Development and verification of the PAM50-based Prosigna breast cancer gene signature assay. BMC Med Genomics.

[25] Shou J, Lai Y, Xu J, Huang J (2016). Prognostic value of FOXA1 in breast cancer: a systematic review and meta-analysis. Breast.

[26] Rangel N, Fortunati N, Osella-Abate S, Annaratone L, Isella C, Catalano MG (2018). FOXA1 and AR in invasive breast cancer: new findings on their co-expression and impact on prognosis in ER-positive patients. BMC Cancer.

[27] Lundgren C, Bendahl PO, Ekholm M, Fernö M, Forsare C, Krüger U (2020). Tumour-infiltrating lymphocytes as a prognostic and tamoxifen predictive marker in premenopausal breast cancer: data from a randomised trial with long-term follow-up. Breast Cancer Res.

[28] Rydén L, Jönsson P-E, Chebil G, Dufmats M, Fernö M, Jirström K (2005). Two years of adjuvant tamoxifen in premenopausal patients with breast cancer: a randomised, controlled trial with long-term follow-up. Eur J Cancer.

[29] Ekholm M, Bendahl P-O, Fernö M, Nordenskjöld B, Stål O, Rydén L (2016). Two years of adjuvant tamoxifen provides a survival benefit compared with no systemic treatment in premenopausal patients with primary breast cancer: long-term follow-up (> 25 years) of the Phase III SBII:2pre trial. J Clin Oncol.

[30] Ekholm M, Bendahl PO, Fernö M, Nordenskjöld B, Stål O, Rydén L (2019). Effects of adjuvant tamoxifen over three decades on breast cancer-free and distant recurrence-free interval among premenopausal women with oestrogen receptor-positive breast cancer randomised in the Swedish SBII:2pre trial. Eur J Cancer.

[31] Gourgou-Bourgade S, Cameron D, Poortmans P, Asselain B, Azria D, Cardoso F (2015). Guidelines for time-to-event end point definitions in breast cancer trials: results of the DATECAN initiative (Definition for the Assessment of Time-to-event Endpoints in CANcer trials). Ann Oncol.

[32] NanoString. nCounter® Breast Cancer 360TM Panel, Product Bulletin. 2020. https://www.nanostring.com/products/ncounter-assays-panels/oncology/breast-cancer-360/. Accessed 15 March 2023.

[33] Benjamini Y, Hochberg Y (1995). Controlling the false discovery rate: a practical and powerful approach to multiple testing. J R Stat Soc B.

[34] Documentation R. Scaling and Centering of Matrix-like Objects. https://stat.ethz.ch/R-manual/R-devel/library/base/html/scale.html. Accessed 20 May 2023.

[35] Wei T Simko V. R package 'corrplot': Visualization of a Correlation Matrix. 2021. https://github.com/taiyun/corrplot. Accessed 20 May 2023.

[36] Gu Z, Eils R, Schlesner M (2016). Complex heatmaps reveal patterns and correlations in multidimensional genomic data. Bioinformatics.

[37] Schoenfeld D (1982). Partial residuals for the proportional hazards regression model. Biometrika.

[38] Altman DG, McShane LM, Sauerbrei W, Taube SE, Taube SE (2012). Reporting recommendations for tumor marker prognostic studies (REMARK): explanation and elaboration. PLoS Med.

[39] McShane LM, Altman DG, Sauerbrei W, Taube SE, Gion M, Clark GM (2005). REporting recommendations for tumour MARKer prognostic studies (REMARK). Br J Cancer.

[40] Schroth W, Büttner FA, Kandabarau S, Hoppe R, Fritz P, Kumbrink J (2020). Gene expression signatures of BRCAness and tumor inflammation define subgroups of early-stage hormone receptor–positive breast cancer patients. Clin Cancer Res.

[41] Cochrane DR, Bernales S, Jacobsen BM, Cittelly DM, Howe EN, D’Amato NC (2014). Role of the androgen receptor in breast cancer and preclinical analysis of enzalutamide. Breast Cancer Res.

[42] Honma N, Horii R, Iwase T, Saji S, Younes M, Ito Y (2014). Proportion of estrogen or progesterone receptor expressing cells in breast cancers and response to endocrine therapy. Breast.

[43] Kuske B, Naughton C, Moore K, Macleod KG, Miller WR, Clarke R (2006). Endocrine therapy resistance can be associated with high estrogen receptor alpha (ERalpha) expression and reduced ERalpha phosphorylation in breast cancer models. Endocr Relat Cancer.

[44] Brett JO, Spring LM, Bardia A, Wander SA (2021). ESR1 mutation as an emerging clinical biomarker in metastatic hormone receptor-positive breast cancer. Breast Cancer Res.

[45] Hurtado A, Holmes KA, Ross-Innes CS, Schmidt D, Carroll JS (2011). FOXA1 is a key determinant of estrogen receptor function and endocrine response. Nat Genet.

[46] Fu X, Jeselsohn R, Pereira R, Hollingsworth EF, Creighton CJ, Li F (2016). FOXA1 overexpression mediates endocrine resistance by altering the ER transcriptome and IL-8 expression in ER-positive breast cancer. Proc Natl Acad Sci U S A.

[47] Ross-Innes CS, Stark R, Teschendorff AE, Holmes KA, Ali HR, Dunning MJ (2012). Differential oestrogen receptor binding is associated with clinical outcome in breast cancer. Nature.

[48] Metovic J, Borella F, D'Alonzo M, Biglia N, Mangherini L, Tampieri C (2022). FOXA1 in breast cancer: a luminal marker with promising prognostic and predictive impact. Cancers (Basel).

[49] Badve S, Turbin D, Thorat MA, Morimiya A, Nielsen TO, Perou CM (2007). FOXA1 expression in breast cancer–correlation with luminal subtype A and survival. Clin Cancer Res.

[50] Tanaka K, Tokunaga E, Yamashita N, Sagara Y, Ohi Y, Taguchi K (2017). The relationship between the expression of FOXA1 and GATA3 and the efficacy of neoadjuvant endocrine therapy. Breast Cancer.

[51] Arruabarrena-Aristorena A, Maag JLV, Kittane S, Cai Y, Karthaus WR, Ladewig E (2020). FOXA1 mutations reveal distinct chromatin profiles and influence therapeutic response in breast cancer. Cancer Cell.

[52] Winter SC, Buffa FM, Silva P, Miller C, Valentine HR, Turley H (2007). Relation of a hypoxia metagene derived from head and neck cancer to prognosis of multiple cancers. Cancer Res.

[53] Buffa FM, Harris AL, West CM, Miller CJ (2010). Large meta-analysis of multiple cancers reveals a common, compact and highly prognostic hypoxia metagene. Br J Cancer.

[54] Rajput AB, Turbin DA, Cheang MC, Voduc DK, Leung S, Gelmon KA (2008). Stromal mast cells in invasive breast cancer are a marker of favourable prognosis: a study of 4444 cases. Breast Cancer Res Treat.

[55] Cai Z, Tang B, Chen L, Lei W (2022). Mast cell marker gene signature in head and neck squamous cell carcinoma. BMC Cancer.

