# Supplementary material for: Tamoxifen-predictive value of gene expression signatures in premenopausal breast cancer: data from the randomized SBII:2 trial
Source: Breast Cancer Res. 2023 Sep 29;25:110. doi: 10.1186/s13058-023-01719-z (PMC10540453; doi:10.1186/s13058-023-01719-z)
Supplement: Supplementary file 1 — Additional file 1. Abbreviated translated study protocol [file 13058_2023_1719_MOESM1_ESM.pdf]

TRANSLATION OF THE SWEDISH PROTOCOL FOR THE SBII:2PRE TRIAL

This protocol also includes the SBII:2post Trial for postmenopausal women and the text regarding that trial has not been translated. There was also a plan for a Radiotherapy Trial, which was never conducted.

One should keep in mind that the SBII:2pre Trial was planned more than 30 years ago in accordance with the contemporary clinical practice.

The current follow-up includes overall mortality and breast cancer-related mortality and the methods and statistics regarding this is described in the submitted manuscript.

SELECTION OF PATIENTS, INCLUDING BOTH ELIGIBILITY AND INELIGIBILITY CRITERIA

Inclusion criteria's

- The patient should have been diagnosed with infiltrating breast cancer
- The patient should be premenopausal, defined as less than 1 year since the last menstruation
- The patient should have undergone modified radical mastectomy or breast conserving therapy
- The patient should have undergone radical surgery
- The patient should not have distant metastasis.

Exclusion criteria's

- Postmenopausal patients, defined as more than 1 year since the last menstruation
- Patients with ongoing pregnancy or lactation
- Patients with breast cancer stage 0, I, III, or IV
- Bilateral breast cancer
- Patients previously treated for malignant tumor disease, except for basal cell carcinoma, cervix cancer and endometrial cancer stage 0
- Patient not expected to cooperate to the extent that the treatment and follow-up require
- Patients that decline to participate

Stratification and randomization

- Randomization will be performed for patients that fulfill the inclusion criteria's and lack all exclusion criteria.
- The patients will be stratified according to the hospital responsible for the surgery.
- The randomization will be performed through telephone contact to the Regional Tumor Register, the University hospital of Lund, Monday to Friday.
- Randomization will be performed in blocks by 8.

SCHEMA AND TREATMENT PLAN, INCLUDING ADMINISTRATION SCHEDULE

- Modified radical surgery according to appendix yy (not available)
- Postoperative radiotherapy according to appendix (not available)

- **Medical treatment**
  - Tamoxifen 20 or 40 mg orally daily for 2 years
  - Control (no adjuvant endocrine therapy)

RULES FOR DOSE MODIFICATION

The trial was performed by two cooperating trial centers. Patients recruited by the South Swedish oncology center (n=427) received 20 mg of tamoxifen while the patients recruited by the South Eastern center (n=137) received 40 mg. (The optimal dose was not known at the time of the trial.)

MEASUREMENT OF TREATMENT PLAN INCLUDING RESPONSE CRITERIA, DEFINITIONS OF RESPONSE AND SURVIVAL, AND METHODS OF MEASUREMENTS

Follow-up

The patients will have follow-up:

- Clinical examination should be performed every 6 month for 5 years
- Chest X-ray and mammogram should be performed annually during 10 years

Evaluation

- Recurrence-free survival
- Site of recurrence
- Toxicity
- Survival

End of treatment

- Patients randomized to tamoxifen should end treatment after 2 years from the start of the treatment.
- The participating centers are notified 2 month before the planned discontinuation of the drug.

REASONS FOR EARLY CESSATION OF THERAPY

- The tamoxifen treatment should be stopped in case of recurrence. It should also be stopped if the patient does not tolerate the drug or if the patient chooses not to continue with the treatment. In all these cases the study secretariat should be informed.
- Patients who stop the treatment without signs of recurrence should continue the follow-up in the same way as those who fulfill the treatment.

OBJECTIVES AND ENTIRE STATISTICAL SECTION

- The study is planned, with a 90% power and an alpha-level of 5%, to detect an absolute difference of 15% regarding 5-year recurrence free survival.
- If in one of the two arms side effects, recurrences or mortality is increased, this group will be discontinued.
- Decisions regarding discontinuation of the study will be taken together by all involved parties.
