# Supplementary figures and images for: Tamoxifen-predictive value of gene expression signatures in premenopausal breast cancer: data from the randomized SBII:2 trial
Source: Breast Cancer Res. 2023 Sep 29;25:110. doi: 10.1186/s13058-023-01719-z (PMC10540453; doi:10.1186/s13058-023-01719-z)

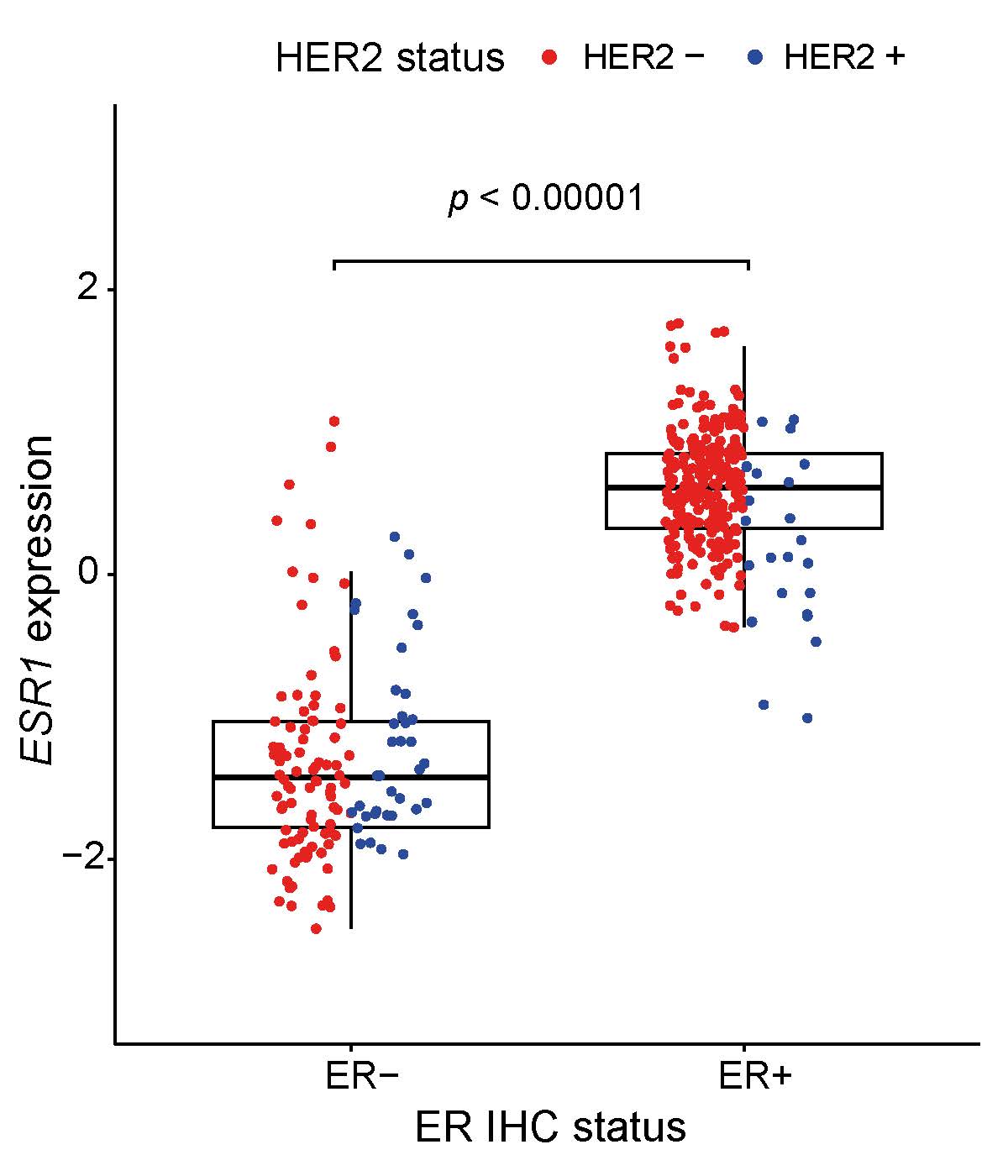

Supplement: Supplementary file 3 — Additional file 3. Correlation between ESR1 gene expression and ER status, based on immunohistochemistry. Abbreviations: ER, estrogen receptor, HER2, human epidermal growth factor receptor 2 [file 13058_2023_1719_MOESM3_ESM.jpg]

All ER+ HER2- tumors

Control - ER+ HER2- tumors

Tam treated - ER+ HER2- tumors

Quartile  
1 2 3 4

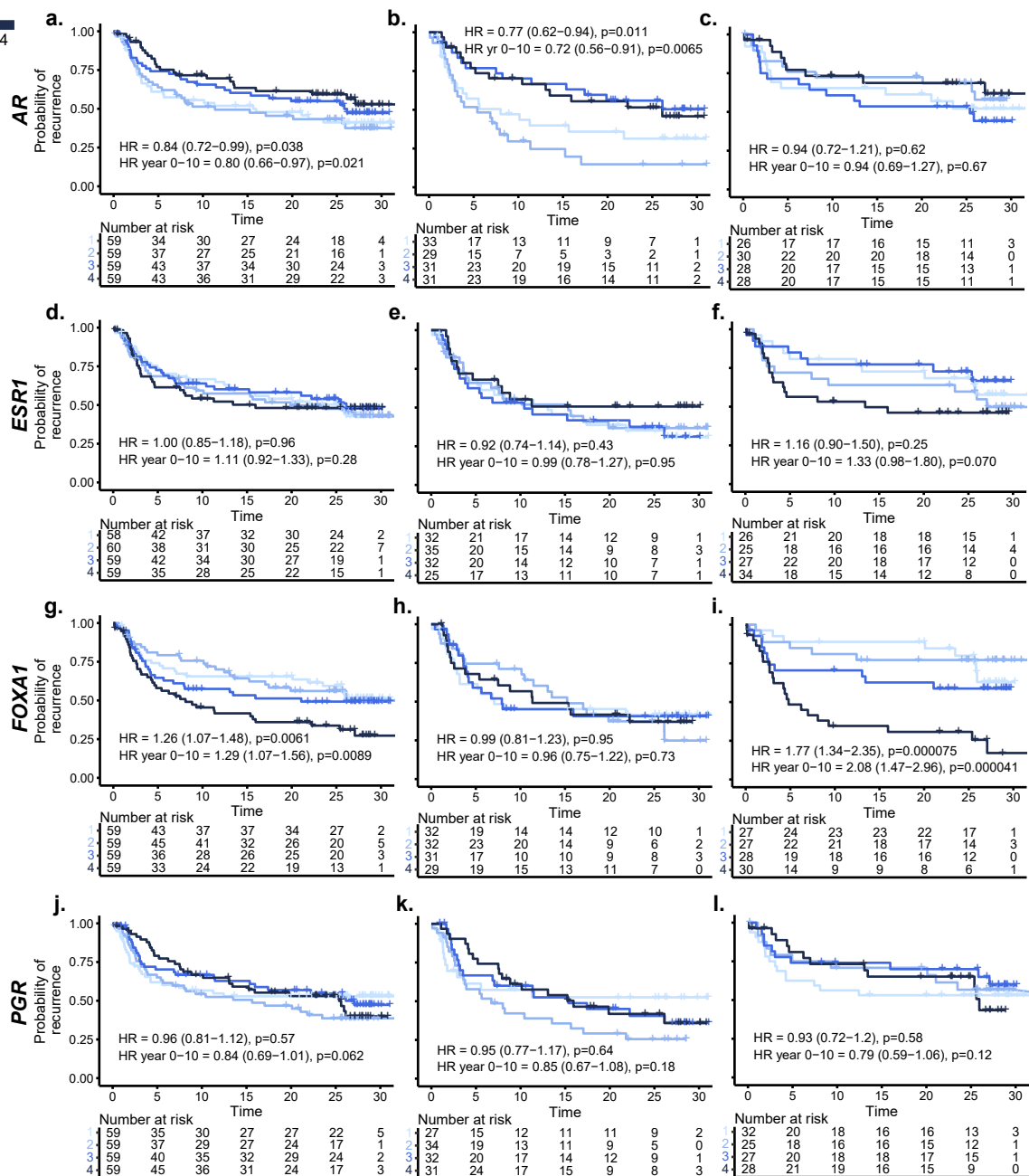

Supplement: Supplementary file 4 — Additional file 4. RFi in relation to quartiles of selected GEX signatures, ER+/HER2− tumors. Kaplan–Meier plots representing the relationship between RFi and GEX levels in terms of quartiles (Q1–Q4) for a–c) AR, d–f) ESR1, g–i) FOXA1, and j–l) PGR in patients with ER+/HER2− tumors (n = 236, left column), ER+/HER2− tumors in the control group (n = 124, middle column), and ER+/HER2− tumors treated with tamoxifen (n = 112, right column). Abbreviations: HER2, human epidermal growth factor receptor 2; HR, hazard ratio; ER, estrogen receptor; RFi, recurrence-free interval [file 13058_2023_1719_MOESM4_ESM.pdf]

Quartile  
1 2 3 4

All ER+ HER2- tumors

Control - ER+ HER2- tumors

Tam treated - ER+ HER2- tumors

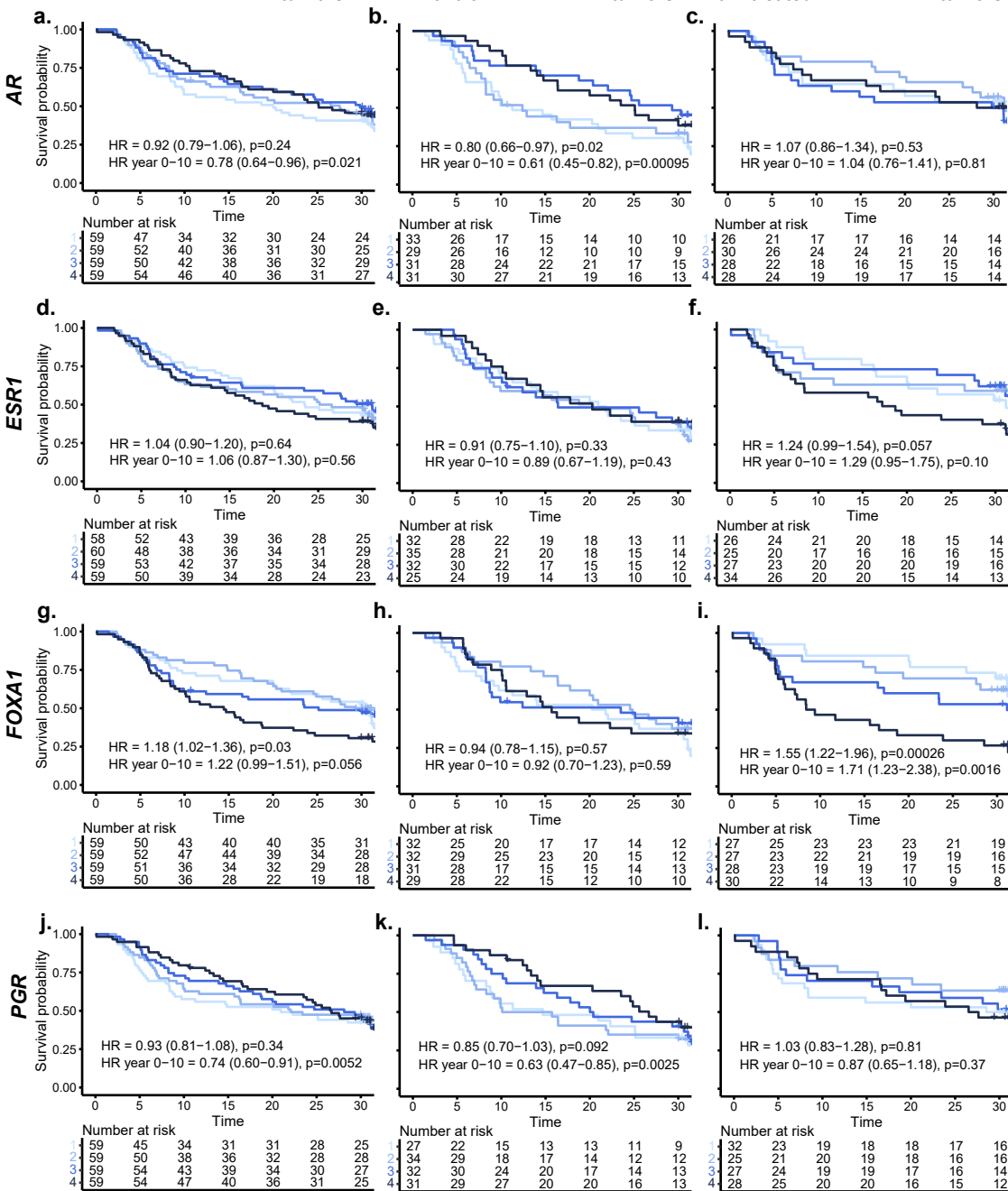

Supplement: Supplementary file 5 — Additional file 5. OS in relation to quartiles of selected GEX signatures, ER+/HER2− tumors. Kaplan–Meier plots representing the relationship between OS and GEX levels in terms of quartiles (Q1–Q4) for a–c) AR, d–f) ESR1, g–i) FOXA1, and j–l) PGR in patients with ER+/HER2− tumors (n = 236, left column), ER+/HER2− tumors in the control group (n = 124, middle column), and ER+/HER2− tumors treated with tamoxifen (n = 112, right column). Abbreviations: HER2, human epidermal growth factor receptor 2; HR, hazard ratio; ER, estrogen receptor; OS, overall survival [file 13058_2023_1719_MOESM5_ESM.pdf]

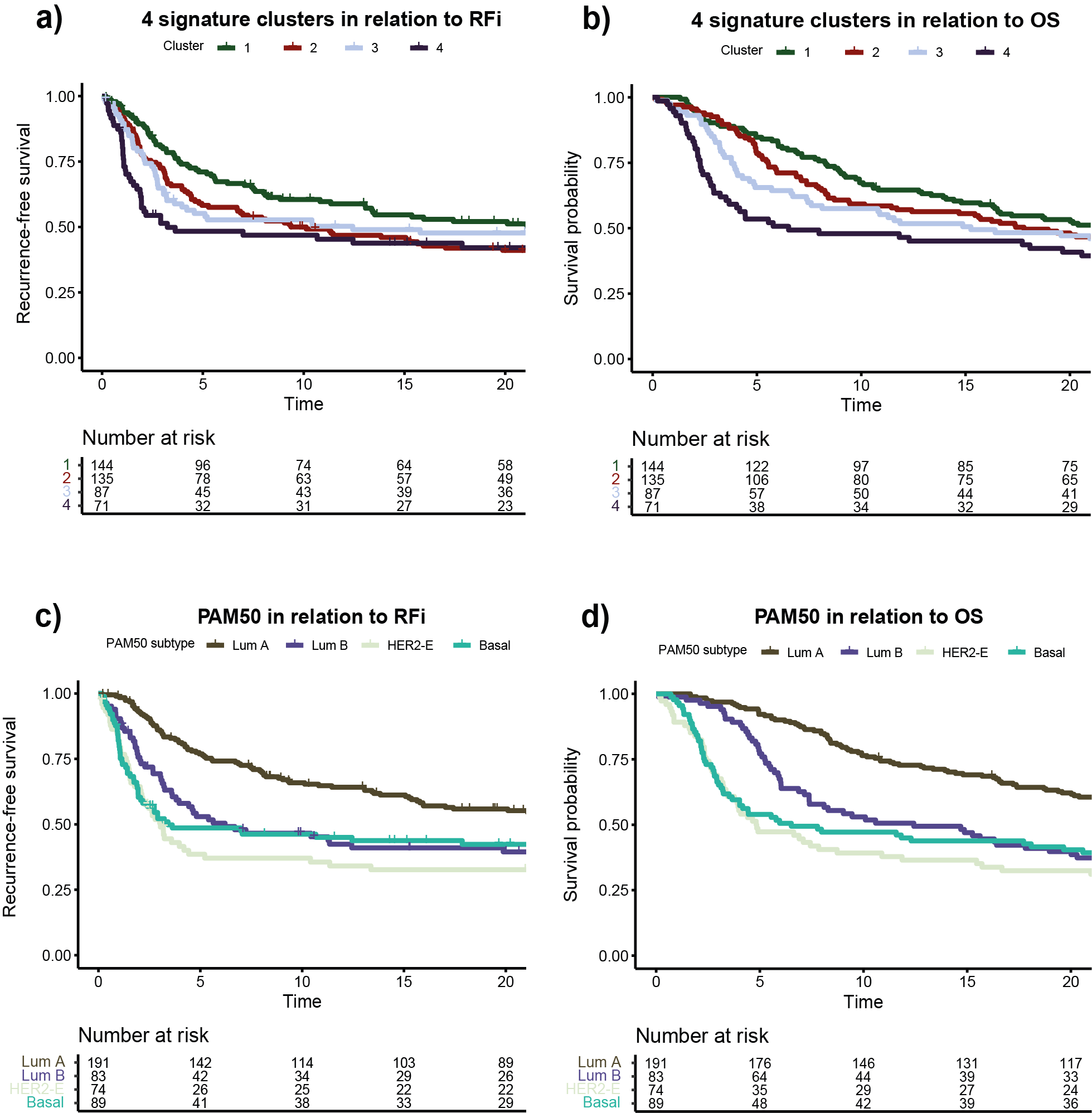

Supplement: Supplementary file 6 — Additional file 6. Outcomes (RFi and OS) of the signature clusters (a–b) and PAM50 subtypes (c–d). Kaplan–Meier plots representing the relationship between four signature clusters (1–4) for the whole cohort (n = 437) for a) RFi and b) OS. Abbreviations: FU, follow-up; HR, hazard ratio; OS, overall survival; RFi, recurrence-free interval [file 13058_2023_1719_MOESM6_ESM.tif]
